# Atrial fibrillation in immigrant groups: a cohort study of all adults 45 years of age and older in Sweden

**DOI:** 10.1007/s10654-017-0283-6

**Published:** 2017-07-12

**Authors:** Per Wändell, Axel C. Carlsson, Xinjun Li, Danijela Gasevic, Johan Ärnlöv, Martin J. Holzmann, Jan Sundquist, Kristina Sundquist

**Affiliations:** 1 Division of Family Medicine and Primary Care, Department of Neurobiology, Care Sciences and Society (NVS), Karolinska Institutet, Huddinge, Sweden; 2 Department of Medical Sciences, Cardiovascular Epidemiology, Uppsala University, Uppsala, Sweden; 3 Center for Primary Health Care Research, Lund University, Malmö, Sweden; 4 Usher Institute of Population Health Sciences and Informatics, College of Medicine and Veterinary Medicine, University of Edinburgh, Edinburgh, UK; 5 School of Health and Social Studies, Dalarna University, Falun, Sweden; 6 Functional Area of Emergency Medicine, Karolinska University Hospital, Stockholm, Sweden; 7 Department of Internal Medicine Solna, Karolinska Institutet, Stockholm, Sweden; 8 Department of Family Medicine and Community Health, Department of Population Health Science and Policy, Icahn School of Medicine at Mount Sinai, New York, NY USA

**Keywords:** Atrial fibrillation, Gender, First generation immigrants, Neighbourhood, Second generation immigrants, Socioeconomic status

## Abstract

To study the association between country of birth and incident atrial fibrillation (AF) in several immigrant groups in Sweden. The study population included all adults (n = 3,226,752) aged 45 years and older in Sweden. AF was defined as having at least one registered diagnosis of AF in the National Patient Register. The incidence of AF in different immigrant groups, using Swedish-born as referents, was assessed by Cox regression, expressed in hazard ratios (HRs) and 95% confidence intervals (CI). All models were stratified by sex and adjusted for age, geographical residence in Sweden, educational level, marital status, and neighbourhood socioeconomic status. Compared to their Swedish-born counterparts, higher incidence of AF [HR (95% CI)] was observed among men from Bosnia 1.74 (1.56–1.94) and Latvia 1.29 (1.09–1.54), and among women from Iraq 1.96 (1.67–2.31), Bosnia 1.88 (1.61–1.94), Finland 1.14 (1.11–1.17), Estonia 1.14 (1.05–1.24) and Germany 1.08 (1.03–1.14). Lower incidence of AF was noted among men (HRs ≤ 0.60) from Iceland, Southern Europe (especially Greece, Italy and Spain), Latin America (especially Chile), Africa, Asia (including Iraq, Turkey, Lebanon and Iran), and among women from Nordic countries (except Finland), Southern Europe, Western Europe (except Germany), Africa, North America, Latin America, Iran, Lebanon and other Asian countries (except Turkey and Iraq). In conclusion, we observed substantial differences in incidence of AF between immigrant groups and the Swedish-born population. A greater awareness of the increased risk of AF development in some immigrant groups may enable for a timely diagnosis, treatment and prevention of its debilitating complications, such as stroke.

**Electronic supplementary material:**

The online version of this article (doi:10.1007/s10654-017-0283-6) contains supplementary material, which is available to authorized users.

## Introduction

Atrial fibrillation (AF) is the most common form of arrhythmia. AF is associated with significant morbidity, in particular with an increased risk of stroke [1, 2]. In 2010, the age-adjusted prevalence of AF worldwide was estimated at approximately 0.6% among men and 0.4% among women [3]. The AF prevalence in Europe was previously estimated to be around 1% [4]. One study conducted in Sweden found the prevalence of a registered diagnosis of AF to be around 2% [5]. As regards to people 20 years of age and older, recent figures show a prevalence of AF in Europe of 2% [6], and in Sweden of 3% [7].

Migration worldwide is on the increase. In Sweden, it is estimated that approximately 17% of the registered Swedish population is foreign-born (data from Statistics Sweden) [8]. The health of immigrants upon arrival to their new country often tends to be better than that of the native population; something that has been termed as the “healthy migrant effect”. This improved health status in some countries could be a result of a selective immigration process in which people are granted entry to a new country if they have passed a medical screening examination. However, such a selection is uncommon for immigrants to Sweden. Additionally, the health of immigrants tends to decline with years spent living in their new adopted country [9, 10]. The relation between immigration and health is complex due to the ethnic, cultural and economic diversity of immigrants. Ethnic differences in the incidence and prevalence of AF [11], as well as in symptoms and treatment [12], have been reported previously. In the United States, a lower AF risk has earlier been reported among Afro-Americans compared to individuals of white European descent [13] and among Chinese and Hispanics compared to non-Hispanic whites [11]. In Europe, AF rates in the UK are lower among South Asians, despite a higher cardiovascular risk profile, than in the native British population [14]. However, there are few other studies on this topic besides those mentioned above.

Therefore, the aim of this study was to explore the risk of being diagnosed with AF among first and second-generation immigrants in Sweden and whether that risk differed from the Swedish-born population, after taking potential confounders into account.

## Methods

### Design

The registers used in the present study were the Total Population Register and the National Patient Register. Sweden’s nationwide population and health care registers have exceptionally high completeness and validity [15]. Less than 1% of the data were missing when linking clinical to national demographic and socioeconomic data. Individuals were tracked using the personal identification numbers, which are assigned to each resident of Sweden. Migrants with a residence permit get a Swedish personal identification number. Asylum seekers are thus not included until they receive their residence permit, but in general they form a limited number of individuals. These identification numbers were replaced with serial numbers to ensure anonymity. Subjects aged 45 years of age and older were included in the study. The follow-up period ran from January 1, 1998 until hospitalisation/out-patient treatment of AF at age of diagnosis of 45 years or more, death, emigration or the end of the study period on December 31, 2012, whichever came first. Out-patient diagnoses were included nationwide from 2001 and onwards from specialist care, not primary health care.

### Study population and co-morbidities

The study included the whole Swedish population aged 45 years and older. Patients with an AF diagnosis prior to January 1, 1998 were excluded in order to “wash-out” those with pre-existing disease. Country of birth was registered and the present study was based on analyses of ten regions (Nordic countries, Southern Europe, Western Europe, Eastern Europe, Baltic countries, Central Europe, Africa, North America, Latin America and Asia) and separate analyses from 27 countries (Supplementary Table 1). Countries with less than 10 observed cases of AF were not analysed separately. First-generation immigrants (n = 434,440) were defined as those born outside Sweden and were compared to Swedish-born individuals. “The date of immigration” is actually the date of residence permit, i.e. when the migrants get their Swedish personal identification number. Second-generation immigrants were defined (n = 121,414) as individuals born in Sweden with at least one foreign-born parent and were compared to individuals born in Sweden with two Swedish-born parents.

Patients with diagnosed AF were identified by the presence of the ICD-10 code (10th version of the World Health Organization’s International Classification of Diseases) for AF (I48) in the National Patient Register. AF diagnosed before 1998, i.e. during the years 1987–1997 (according to ICD-9 1987–1996 and ICD-10 1997) were excluded. We also identified co-morbidities according to ICD-10 for the following diagnoses: hypertension I10-I19, chronic rheumatic heart disease I05-I09, CHD I20-I25, heart failure I50, stroke I60-I69, diabetes E10-E14, obesity E65-E68, alcoholism and related disorders F10 and K70, and chronic obstructive pulmonary disease (COPD) J40-J47.

### Outcome variable

Time was calculated from January 1, 1998 until hospitalisation/out-patient treatment of AF (among individuals at an age of diagnosis of 45 years or older), death, emigration or the end of the study period on December 31, 2012, whichever came first.

### Demographic and socioeconomic variables

The study population was stratified by *sex*.

*Age* was used as a continuous variable in the analysis.

*Educational attainment* was categorised as ≤9 years (partial or complete compulsory schooling), 10–12 years (partial or complete secondary schooling) and >12 years (attendance at college and/or university).

*Geographic region of residence* was included in order to adjust for possible regional differences in hospital admissions and was categorised as [1] large cities, [2] southern Sweden and [3] northern Sweden. Large cities were defined as municipalities with a population of >200,000 and comprised the three largest cities in Sweden: Stockholm, Gothenburg and Malmö.

### Neighbourhood socioeconomic status

Neighbourhoods were derived from Small Area Market Statistics (SAMS). These were originally created for commercial purposes and pertain to small geographic areas with boundaries defined by homogenous types of buildings. The average population in each SAMS neighbourhood is approximately 2000 people for Stockholm and 1000 people for the rest of Sweden. A summary index was calculated to characterise neighbourhood-level deprivation. The neighbourhood index was based on information about female and male residents aged 20–64 years. The logic for this is because this age group represents those who are among the most socioeconomically active in the population (i.e. a group that has a stronger impact on the socioeconomic structure in the neighbourhood compared to children, younger women and men, and retirees). The index was based on the following four variables: low educational status (<10 years of formal education); income from all sources, including interest and dividends, that is <50% of the median individual income); unemployment (excluding full-time students, those completing military service and early retirees); and receipt of social welfare. The index was categorised into three groups: more than one standard deviation (SD) below the mean (high SES or low-deprivation level), more than one SD above the mean (low SES or high-deprivation level), and within one SD of the mean (middle SES or middle-deprivation level) [16], with neighbourhood status classified as high, middle or low SES, corresponding to the categories low, middle and high-deprivation in the index [17].

### Statistical analysis

The number of AF cases was presented for first- and second-generation immigrants and across baseline subject characteristics. Cox regression analysis was used for estimating the risk of incident AF in different immigrant groups compared to the Swedish-born population. All analyses were stratified by sex. Three models were used in our analyses: Model 1 was adjusted for age and region of residence in Sweden; Model 2 was adjusted for age, region of residence in Sweden, educational level, marital status and neighbourhood SES; Model 3 was constructed as Model 2 with inclusion of co-morbidities. In addition, Cox regression sensitivity analyses were performed in which first-generation immigrants moving to Sweden within the last 5 years of follow-up were excluded.

The study was approved by the regional ethics boards at Karolinska Institutet and Lund University.

## Results

Table 1 features the characteristics of the included samples for analysis for first- and second-generation immigrants 45 years of age and above. There were 9.4% of people diagnosed with AF among first-generation immigrants; 5.7% for second-generation immigrants. AF was less common among immigrants in general compared to Swedish-born individuals. AF was also less common among females, individuals with a higher level of formal education, married individuals, and people living in northern Sweden, while AF was more common among individuals with co-morbidities, especially cardiovascular co-morbidities.

**Table 1:** Population and number of incident cases of atrial fibrillation (AF) diagnoses in the Swedish population, used to study AF in first-generation and second-generation immigrants compared to Swedish-born individuals

	First-generation analysis				Second-generation analysis			
	Population		AF diagnosis		Population		AF diagnosis	
	No	%	No.	%	No	%	No.	%
Total population	3,226,752		304,487		1,890,853		107,213	
Gender								
Males	1,520,562	47.1	159,769	52.5	950,316	50.3	70,631	65.9
Females	1,706,190	52.9	144,718	47.5	940,537	49.7	36,582	34.1
Country of origin^a^								
Sweden	2,792,312	86.5	278,472	91.5	1,769,439	93.6	101,985	95.1
Other countries	434,440	13.5	26,015	8.5	121,414	6.4	5228	4.9
Birth year								
–1909	73,467	2.3	4945	1.6				
1910–1919	323,206	10.0	51,267	16.8				
1920–1929	602,445	18.7	106,131	34.9				
1930-39	722,107	22.4	79,921	26.2	441,357	23.3	45,472	42.4
1940–1949	1,088,739	33.7	52,610	17.3	1,034,701	54.7	51,589	48.1
1950–	416,788	12.9	9613	3.2	414,795	21.9	10,152	9.5
Educational level								
≤9	1,511,090	46.8	162,796	53.5	606,689	32.1	38,917	36.3
10–12	811,538	25.2	68,676	22.6	569,256	30.1	29,557	27.6
>12	904,124	28.0	73,015	24.0	714,908	37.8	38,739	36.1
Region of residence								
Large cities	1,075,763	33.3	110,525	36.3	642,798	34.0	38,166	35.6
Southern Sweden	1,424,349	44.1	140,915	46.3	868,490	45.9	49,178	45.9
Northern Sweden	726,640	22.5	53,047	17.4	379,565	20.1	19,869	18.5
Marital status								
Married	2,612,169	81.0	235,927	77.5	1,515,664	80.2	82,165	76.6
Unmarried	614,583	19.0	68,560	22.5	375,189	19.8	25,048	23.4
Neighbourhood deprivation								
Low	485,193	15.0	43,082	14.1	337,143	17.8	18,330	17.1
Middle	1,622,097	50.3	164,111	53.9	971,071	51.4	56,101	52.3
High	359,648	11.1	36,422	12.0	194,895	10.3	11,617	10.8
Unknown	759,814	23.5	60,872	20.0	387,744	20.5	21,165	19.7
Hospital diagnosis of COPD								
No	3,024,792	93.7	271,514	89.2	1,793,433	94.8	96,629	90.1
Yes	201,960	6.3	32,973	10.8	97,420	5.2	10,584	9.9
Hospital diagnosis of obesity								
No	3,199,756	99.2	300,514	98.7	1,868,020	98.8	104,092	97.1
Yes	26,996	0.8	3973	1.3	22,833	1.2	3121	2.9
Hospital diagnosis of CHD								
No	2,747,889	85.2	203,705	66.9	1,705,792	90.2	79,507	74.2
Yes	478,863	14.8	100,782	33.1	185,061	9.8	27,706	25.8
Hospital diagnosis of diabetes								
No	2,942,192	91.2	259,615	85.3	1,746,278	92.4	90,514	84.4
Yes	284,560	8.8	44,872	14.7	144,575	7.6	16,699	15.6
Hospital diagnosis of alcoholism and related disorders								
No	3,160,834	98.0	297,828	97.8	1,832,992	96.9	102,324	95.4
Yes	65,918	2.0	6659	2.2	57,861	3.1	4889	4.6
Hospital diagnosis of stroke								
No	2,895,083	89.7	227,755	74.8	1,784,381	94.4	90,851	84.7
Yes	331,669	10.3	76,732	25.2	106,472	5.6	16,362	15.3
Hospital diagnosis of hypertension								
No	2,573,075	79.7	180,959	59.4	1,542,546	81.6	56,754	52.9
Yes	653,677	20.3	123,528	40.6	348,307	18.4	50,459	47.1
Hospital diagnosis of heart failure								
No	2,939,005	91.1	194,225	63.8	1,826,817	96.6	82,429	76.9
Yes	287,747	8.9	110,262	36.2	64,036	3.4	24,784	23.1
Hospital diagnosis of chronic rheumatic heart disease								
No	3,220,896	99.8	301,799	99.1	1,888,802	99.9	106,320	99.2
Yes	5856	0.2	2688	0.9	2051	0.1	893	0.8

^a^Immigrant status in second generation was based on parental birth country

Table 2a, b show the incidence of AF in first generation male and female immigrants, respectively, compared to their Swedish-born counterparts. In comparison to Swedish-born men, the incidence of AF was higher among male immigrants with Bosnian origin, after adjustment for age, region of residence in Sweden, educational level, marital status, neighbourhood SES and co-morbidity. By contrast, compared to Swedish-born men, the incidence of AF was lower in men originating from most other regions and countries, but especially low (HRs ≤ 0.60) among immigrant men from Iceland, from Southern Europe (especially Greece, Italy and Spain), Latin America (especially Chile), Africa, Asia and specifically Iraq, Turkey, Lebanon and Iran. Compared to Swedish-born women, the incidence of AF was higher among immigrant women from Bosnia and Iraq and there was a borderline significant increase among women from Finland and Estonia. A lower incidence of AF was observed among immigrant women from most other regions and countries and was especially low for women (HRs ≤ 0.60) from Iceland, Greece, Italy, Africa, and Latin America.

**Table 2:** Incidence of [hazard ratio (HR) with 95% confidence intervals (95% CI)] AF in (a) first-generation male immigrants compared to Swedish-born (N = 1,520,562), (b) first-generation female immigrants compared to Swedish-born individuals (N = 1,706,190)

	Model 1			Model 2			Model 3		
	HR	95% CI		HR	95% CI		HR	95% CI	
*(a)*									
Sweden	1			1			1		
Nordic countries	**0.76**	**0.74**	**0.78**	**0.85**	**0.83**	**0.87**	**0.83**	**0.81**	**0.86**
Denmark	**0.68**	**0.64**	**0.72**	**0.70**	**0.66**	**0.74**	**0.71**	**0.67**	**0.75**
Finland	**0.78**	**0.76**	**0.81**	**0.91**	**0.88**	**0.94**	**0.87**	**0.85**	**0.90**
Iceland	**0.24**	**0.16**	**0.36**	**0.29**	**0.20**	**0.44**	**0.34**	**0.22**	**0.50**
Norway	**0.80**	**0.75**	**0.85**	**0.86**	**0.81**	**0.91**	**0.87**	**0.82**	**0.93**
Southern Europe	**0.37**	**0.34**	**0.40**	**0.44**	**0.40**	**0.48**	**0.48**	**0.44**	**0.52**
France	**0.53**	**0.41**	**0.67**	**0.62**	**0.49**	**0.79**	**0.66**	**0.52**	**0.84**
Greece	**0.28**	**0.24**	**0.33**	**0.35**	**0.30**	**0.41**	**0.40**	**0.34**	**0.46**
Italy	**0.44**	**0.38**	**0.51**	**0.52**	**0.45**	**0.60**	**0.53**	**0.46**	**0.61**
Spain	**0.36**	**0.28**	**0.45**	**0.44**	**0.34**	**0.55**	**0.48**	**0.38**	**0.61**
Other Southern Europe	**0.34**	**0.25**	**0.48**	**0.36**	**0.26**	**0.50**	**0.41**	**0.29**	**0.57**
Western Europe	**0.70**	**0.67**	**0.74**	**0.76**	**0.73**	**0.80**	**0.77**	**0.74**	**0.81**
The Netherlands	**0.63**	**0.52**	**0.77**	**0.69**	**0.57**	**0.83**	**0.72**	**0.59**	**0.87**
UK and Ireland	**0.46**	**0.39**	**0.53**	**0.54**	**0.47**	**0.63**	**0.61**	**0.52**	**0.71**
Germany	**0.77**	**0.72**	**0.82**	**0.81**	**0.76**	**0.86**	**0.80**	**0.76**	**0.85**
Austria	**0.77**	**0.68**	**0.88**	**0.84**	**0.74**	**0.95**	**0.83**	**0.73**	**0.94**
Other Western Europe	**0.59**	**0.47**	**0.75**	**0.69**	**0.54**	**0.87**	**0.72**	**0.57**	**0.91**
Eastern Europe	**0.75**	**0.71**	**0.79**	**0.78**	**0.74**	**0.83**	**0.78**	**0.74**	**0.83**
Bosnia	**1.28**	**1.12**	**1.46**	**1.80**	**1.58**	**2.06**	**1.48**	**1.30**	**1.69**
Yugoslavia	**0.68**	**0.64**	**0.73**	**0.70**	**0.65**	**0.75**	**0.71**	**0.66**	**0.76**
Croatia	**0.73**	**0.58**	**0.92**	**0.68**	**0.55**	**0.86**	**0.73**	**0.58**	**0.92**
Romania	**0.75**	**0.62**	**0.90**	**0.76**	**0.63**	**0.92**	**0.75**	**0.62**	**0.90**
Bulgaria	**0.60**	**0.42**	**0.86**	**0.65**	**0.45**	**0.93**	0.70	0.49	1.00
Other Eastern Europe	**0.71**	**0.50**	**1.01**	**0.64**	**0.45**	**0.91**	**0.67**	**0.47**	**0.96**
Baltic countries	1.05	0.97	1.14	**1.10**	**1.02**	**1.20**	1.07	0.99	1.16
Estonia	1.03	0.94	1.12	1.08	0.99	1.18	1.05	0.96	1.15
Latvia	1.17	0.96	1.42	1.21	0.99	1.47	1.19	0.98	1.44
Central Europe	**0.79**	**0.74**	**0.84**	**0.79**	**0.75**	**0.84**	**0.75**	**0.71**	**0.80**
Poland	**0.76**	**0.69**	**0.84**	**0.77**	**0.70**	**0.85**	**0.73**	**0.67**	**0.81**
Other Central Europe	**0.80**	**0.70**	**0.92**	**0.81**	**0.71**	**0.93**	**0.78**	**0.68**	**0.89**
Hungary	**0.81**	**0.73**	**0.89**	**0.80**	**0.73**	**0.88**	**0.76**	**0.69**	**0.84**
Africa	**0.41**	**0.36**	**0.48**	**0.48**	**0.42**	**0.56**	**0.51**	**0.43**	**0.59**
North America	**0.61**	**0.54**	**0.69**	**0.69**	**0.61**	**0.78**	**0.72**	**0.64**	**0.82**
Latin America	**0.29**	**0.25**	**0.34**	**0.34**	**0.29**	**0.39**	**0.38**	**0.32**	**0.45**
Chile	**0.26**	**0.21**	**0.32**	**0.30**	**0.24**	**0.37**	**0.33**	**0.27**	**0.42**
South America	**0.34**	**0.27**	**0.43**	**0.39**	**0.31**	**0.49**	**0.45**	**0.36**	**0.57**
Asia	**0.48**	**0.45**	**0.51**	**0.55**	**0.52**	**0.59**	**0.53**	**0.49**	**0.56**
Turkey	**0.47**	**0.41**	**0.54**	**0.56**	**0.49**	**0.65**	**0.53**	**0.46**	**0.61**
Lebanon	**0.41**	**0.30**	**0.55**	**0.48**	**0.36**	**0.65**	**0.43**	**0.32**	**0.58**
Iran	**0.39**	**0.33**	**0.45**	**0.41**	**0.35**	**0.48**	**0.41**	**0.35**	**0.48**
Iraq	**0.80**	**0.68**	**0.92**	1.00	0.86	1.16	**0.83**	**0.71**	**0.96**
Other Asia countries	**0.45**	**0.40**	**0.51**	**0.52**	**0.47**	**0.59**	**0.51**	**0.46**	**0.58**
Russia	1.07	0.93	1.23	1.11	0.97	1.28	1.01	0.88	1.16
*(b)*									
Sweden	1			1			1		
Nordic countries	**0.96**	**0.94**	**0.98**	**1.01**	**0.99**	**1.04**	**0.97**	**0.95**	**0.99**
Denmark	**0.78**	**0.73**	**0.83**	**0.79**	**0.74**	**0.84**	**0.81**	**0.75**	**0.86**
Finland	1.02	0.99	1.05	**1.10**	**1.07**	**1.13**	**1.03**	**1.00**	**1.06**
Iceland	**0.37**	**0.24**	**0.57**	**0.43**	**0.28**	**0.66**	**0.50**	**0.32**	**0.78**
Norway	**0.91**	**0.86**	**0.96**	**0.92**	**0.87**	**0.97**	**0.89**	**0.85**	**0.94**
Southern Europe	**0.42**	**0.37**	**0.48**	**0.48**	**0.43**	**0.54**	**0.55**	**0.49**	**0.62**
France	**0.62**	**0.46**	**0.82**	**0.72**	**0.54**	**0.96**	0.79	0.59	1.06
Greece	**0.30**	**0.24**	**0.38**	**0.35**	**0.28**	**0.44**	**0.42**	**0.33**	**0.53**
Italy	**0.38**	**0.30**	**0.48**	**0.44**	**0.35**	**0.56**	**0.49**	**0.39**	**0.62**
Spain	**0.61**	**0.46**	**0.82**	**0.70**	**0.52**	**0.94**	0.77	0.58	1.03
Other Southern Europe	**0.57**	**0.38**	**0.87**	**0.59**	**0.39**	**0.90**	0.66	0.44	1.00
Western Europe	**0.90**	**0.86**	**0.95**	0.97	0.92	1.02	0.96	0.91	1.01
The Netherlands	**0.72**	**0.56**	**0.93**	0.80	0.62	1.03	0.85	0.66	1.10
UK and Ireland	**0.48**	**0.39**	**0.59**	**0.55**	**0.45**	**0.68**	**0.61**	**0.50**	**0.76**
Germany	0.98	0.93	1.04	1.04	0.98	1.10	1.01	0.95	1.06
Austria	0.96	0.82	1.12	1.03	0.88	1.20	1.01	0.87	1.18
Other Western Europe	**0.62**	**0.46**	**0.85**	**0.72**	**0.53**	**0.98**	0.75	0.55	1.03
Eastern Europe	0.96	0.89	1.03	0.98	0.91	1.05	0.97	0.90	1.04
Bosnia	**1.54**	**1.27**	**1.87**	**2.03**	**1.67**	**2.46**	**1.67**	**1.38**	**2.03**
Yugoslavia	**0.90**	**0.82**	**0.98**	**0.89**	**0.82**	**0.98**	**0.90**	**0.82**	**0.99**
Croatia	1.05	0.78	1.41	0.99	0.74	1.34	1.06	0.79	1.43
Romania	0.81	0.63	1.04	0.86	0.67	1.11	0.80	0.62	1.03
Bulgaria	0.83	0.51	1.33	0.95	0.59	1.52	0.95	0.59	1.53
Other Eastern Europe	1.09	0.63	1.87	0.98	0.57	1.69	0.98	0.57	1.69
Baltic countries	1.05	0.97	1.14	**1.13**	**1.05**	**1.23**	1.08	0.99	1.17
Estonia	1.07	0.98	1.16	**1.15**	**1.05**	**1.25**	**1.10**	**1.00**	**1.20**
Latvia	0.96	0.77	1.20	1.05	0.84	1.30	0.97	0.78	1.21
Central Europe	**0.90**	**0.84**	**0.97**	**0.93**	**0.87**	**1.00**	**0.87**	**0.81**	**0.94**
Poland	**0.91**	**0.83**	**1.01**	**0.94**	**0.85**	**1.04**	**0.86**	**0.78**	**0.95**
Other Central Europe	**0.77**	**0.65**	**0.91**	**0.81**	**0.69**	**0.96**	**0.81**	**0.68**	**0.95**
Hungary	0.98	0.87	1.11	1.01	0.90	1.15	0.93	0.82	1.05
Africa	**0.47**	**0.33**	**0.68**	**0.54**	**0.38**	**0.78**	**0.55**	**0.38**	**0.80**
North America	**0.80**	**0.71**	**0.90**	**0.87**	**0.77**	**0.98**	0.89	0.79	1.01
Latin America	**0.41**	**0.33**	**0.50**	**0.45**	**0.37**	**0.55**	**0.48**	**0.39**	**0.59**
Chile	**0.42**	**0.32**	**0.55**	**0.46**	**0.35**	**0.61**	**0.48**	**0.37**	**0.64**
South America	**0.39**	**0.29**	**0.54**	**0.44**	**0.32**	**0.60**	**0.48**	**0.35**	**0.65**
Asia	**0.81**	**0.75**	**0.89**	**0.89**	**0.81**	**0.97**	**0.83**	**0.76**	**0.91**
Turkey	0.92	0.79	1.08	1.00	0.86	1.17	0.89	0.77	1.04
Lebanon	0.69	0.45	1.06	0.75	0.49	1.14	**0.61**	**0.40**	**0.94**
Iran	**0.72**	**0.57**	**0.91**	**0.76**	**0.60**	**0.96**	**0.76**	**0.60**	**0.95**
Iraq	**1.55**	**1.23**	**1.96**	**1.91**	**1.51**	**2.41**	**1.52**	**1.20**	**1.92**
Other Asian countries	**0.65**	**0.56**	**0.76**	**0.71**	**0.61**	**0.83**	**0.71**	**0.61**	**0.83**
Russia	0.98	0.85	1.14	1.03	0.89	1.19	0.92	0.80	1.07

Model 1 was adjusted for age and region of residence in Sweden; Model 2 was adjusted for age, region of residence in Sweden, educational level, marital status and neighbourhood SES; Model 3 was constructed as Model 2 with inclusion of co-morbidities

Significant values HRs and 95% CIs marked by bold

Table 3a, b show the incidence of AF in the second-generation male and female immigrants, respectively, compared to their Swedish-born counterparts. An increased incidence of AF was found only in the fully adjusted model among males from the Netherlands and not among any female immigrant groups, after adjusting for age, region of residence, educational level, marital status, neighbourhood SES and co-morbidity. Second-generation male immigrants from Italy and Latin America had a lower incidence (with HRs ≤ 0.60) of AF compared to their Swedish-born counterparts. Among second-generation immigrant women, compared to Swedish-born women, a lower incidence of AF (with HRs ≤ 0.60) was observed for those women with origin from Southern Europe, especially from Italy.

**Table 3:** Incidence of [hazard ratio (HR) with 95% confidence intervals (95% CI)] AF in (a) second-generation male immigrants compared to Swedish-born individuals (N = 950,316), (b) second-generation female immigrants compared to Swedish-born individuals (N = 940,537)

	Model 1			Model 2			Model 3		
	HR	95% CI		HR	95% CI		HR	95% CI	
*(a)*									
Sweden	1			1			1		
Nordic countries	0.97	0.93	1.02	0.98	0.94	1.02	0.96	0.91	1.00
Denmark	0.95	0.86	1.05	0.95	0.86	1.05	0.97	0.88	1.07
Finland	0.99	0.93	1.06	1.00	0.94	1.06	0.96	0.90	1.02
Norway	0.96	0.89	1.04	0.96	0.89	1.04	0.94	0.87	1.02
Southern Europe	0.78	0.60	1.02	0.80	0.61	1.04	0.82	0.63	1.07
Italy	0.73	0.50	1.06	0.74	0.51	1.08	0.76	0.52	1.11
Western Europe	**0.90**	**0.82**	**0.99**	**0.90**	**0.82**	**0.99**	**0.90**	**0.82**	**0.99**
The Netherlands	1.37	0.95	1.99	1.38	0.95	2.00	**1.47**	**1.01**	**2.13**
UK and Ireland	1.11	0.83	1.48	1.12	0.84	1.49	1.12	0.84	1.50
Germany	**0.86**	**0.76**	**0.96**	**0.86**	**0.77**	**0.96**	**0.85**	**0.76**	**0.95**
Austria	0.86	0.65	1.14	0.86	0.65	1.15	0.84	0.63	1.12
Other Western Europe	0.78	0.45	1.38	0.79	0.45	1.39	0.92	0.52	1.62
Eastern Europe	0.88	0.63	1.22	0.88	0.64	1.22	0.84	0.61	1.17
Yugoslavia	0.85	0.56	1.29	0.85	0.56	1.30	0.80	0.52	1.21
Romania	0.85	0.46	1.58	0.86	0.46	1.60	0.90	0.48	1.67
Baltic countries	0.97	0.87	1.09	0.98	0.87	1.10	0.99	0.89	1.11
Estonia	0.95	0.84	1.07	0.95	0.84	1.08	0.98	0.86	1.11
Latvia	1.14	0.87	1.51	1.15	0.87	1.52	1.10	0.84	1.45
Central Europe	**0.79**	**0.66**	**0.94**	**0.79**	**0.66**	**0.94**	**0.75**	**0.63**	**0.89**
Poland	0.86	0.68	1.09	0.86	0.68	1.09	0.83	0.65	1.04
Other Central Europe	1.03	0.75	1.40	1.03	0.75	1.40	0.98	0.72	1.33
Hungary	**0.40**	**0.25**	**0.66**	**0.40**	**0.25**	**0.66**	**0.38**	**0.23**	**0.61**
Africa	0.42	0.13	1.29	0.42	0.14	1.30	0.47	0.15	1.44
North America	0.93	0.83	1.05	0.94	0.83	1.06	0.96	0.85	1.09
Latin America	0.59	0.27	1.31	0.60	0.27	1.33	0.60	0.27	1.34
Asia	0.65	0.42	1.02	0.66	0.42	1.04	0.68	0.43	1.06
Other Asian countries	0.84	0.52	1.35	0.85	0.53	1.37	0.92	0.58	1.49
Russia	1.12	0.94	1.34	1.12	0.94	1.35	1.11	0.93	1.34
*(b)*									
Sweden	1			1			1		
Nordic countries	1.04	0.98	1.10	1.04	0.98	1.11	0.99	0.93	1.05
Denmark	0.98	0.85	1.13	0.97	0.84	1.13	0.99	0.86	1.15
Finland	**1.12**	**1.02**	**1.22**	**1.12**	**1.03**	**1.22**	1.03	0.94	1.12
Norway	0.96	0.86	1.07	0.96	0.86	1.07	0.93	0.84	1.04
Southern Europe	**0.51**	**0.31**	**0.84**	**0.53**	**0.33**	**0.87**	**0.58**	**0.36**	**0.95**
Italy	**0.24**	**0.09**	**0.64**	**0.25**	**0.09**	**0.67**	**0.28**	**0.11**	**0.75**
Western Europe	**0.84**	**0.73**	**0.97**	**0.86**	**0.75**	**0.99**	0.91	0.79	1.05
The Netherlands	0.87	0.43	1.74	0.89	0.44	1.77	1.01	0.51	2.02
UK and Ireland	0.88	0.54	1.41	0.90	0.56	1.45	0.97	0.60	1.56
Germany	**0.82**	**0.70**	**0.97**	**0.84**	**0.71**	**0.99**	0.88	0.74	1.04
Austria	0.90	0.61	1.34	0.92	0.62	1.37	0.99	0.67	1.46
Other Western Europe	0.96	0.46	2.02	1.01	0.48	2.12	1.14	0.55	2.40
Eastern Europe	0.56	0.29	1.07	0.56	0.29	1.07	0.54	0.28	1.05
Yugoslavia	0.39	0.15	1.03	0.38	0.14	1.01	0.39	0.15	1.05
Romania	0.87	0.33	2.31	0.90	0.34	2.39	0.79	0.30	2.10
Baltic countries	0.90	0.77	1.06	0.92	0.78	1.09	0.91	0.78	1.08
Estonia	0.88	0.74	1.05	0.90	0.75	1.08	0.88	0.74	1.06
Latvia	1.03	0.69	1.54	1.06	0.71	1.58	1.12	0.75	1.67
Central Europe	**0.71**	**0.54**	**0.93**	**0.72**	**0.55**	**0.94**	**0.68**	**0.52**	**0.90**
Poland	0.83	0.58	1.19	0.83	0.58	1.19	0.79	0.55	1.12
Other Central Europe	**0.42**	**0.21**	**0.83**	**0.42**	**0.21**	**0.84**	**0.41**	**0.20**	**0.82**
Hungary	0.79	0.47	1.34	0.80	0.47	1.35	0.77	0.46	1.30
Africa	0.90	0.29	2.79	0.93	0.30	2.89	1.27	0.41	3.95
North America	0.84	0.70	1.00	0.84	0.71	1.00	0.87	0.73	1.04
Latin America	0.48	0.12	1.92	0.49	0.12	1.94	0.60	0.15	2.38
Asia	0.50	0.22	1.11	0.51	0.23	1.14	0.53	0.24	1.18
Other Asian countries	0.57	0.24	1.36	0.59	0.25	1.42	0.64	0.26	1.53
Russia	1.04	0.81	1.34	1.05	0.82	1.36	0.99	0.77	1.28

Model 1 was adjusted for age and region of residence in Sweden; Model 2 was adjusted for age, region of residence in Sweden, educational level, marital status and neighbourhood SES; Model 3 was constructed as Model 2 with inclusion of co-morbidities

Significant values HRs and 95% CIs marked by bold

The results of the sensitivity analyses performed in first-generation immigrants (Supplementary Table 2) confirmed the results from Table 2a, b, and only small differences were observed.

## Discussion

This study explored the risk of being diagnosed with incident AF among first and second-generation immigrant men and women in Sweden compared to Swedish-born men and women aged 45 years and older. Both higher and lower estimates of AF were detected in the different immigrant groups. Higher estimates were found among first-generation immigrants for both men and women from Bosnia and among women from Iraq, while no excess incidence of AF was found among second-generation immigrants. Furthermore, compared to Swedes, lower incidence of AF was found in first-generation immigrants for most other immigrant groups, while this was true only for a few groups of the second-generation immigrant groups, i.e. among men from Germany and Hungary, and women from Italy and Central Europe.

AF prevalence differs around the world and some differences are to be expected, in particular a lower AF incidence among immigrants of non-European descent from non-Western countries [18]. This could explain the lower incidence among immigrants from certain regions, such as Africa, Latin America and many Asian countries. Furthermore, individuals living in Northern Europe traditionally have a higher risk of coronary heart disease (CHD), especially compared to Southern Europe. As myocardial infarction is a risk factor for AF [19], the lower AF incidence among most immigrant groups could reflect a higher AF incidence and prevalence in Sweden [6].

There were some findings that could not be easily interpreted. For instance, the AF incidence differed among immigrants from different regions of Europe, with lower incidence among immigrants from most European regions. However, there were some important exceptions, in particular the increased incidence among men and women from Bosnia. Risk factors for AF include older age, sex, genetics, hypertension, heart disease (heart failure and coronary artery disease), being overweight and obese, higher amount of pericardial fat, sleep apnea, atrial dilatation and stretch, chronic kidney disease, smoking, high alcohol consumption, diabetes and thyroid dysfunction [20]. The risk pattern differs in different immigrant groups and could contribute to, and possibly explain, differences in AF incidence. We were able to adjust for some co-morbidities, especially cardiovascular co-morbidity, but not for other clinical factors. Some ethnic differences in the risk factor pattern for AF have been shown, i.e. hypertension [21], diabetes [22], smoking [23] and obesity [22]. Even if hypertension is the most commonly established risk factor for AF worldwide, other factors such as rheumatic and valvular heart diseases seem to be more important for AF in populations living in Latin America, India, the Middle East and Africa [24]. On a global scale, hypertension is more prevalent in Central and Eastern Europe and also in Sub-Saharan Africa and South Asia [25] but we found no increased AF incidence among most Central or Eastern European immigrants. In Europe, hypertension is more common among immigrants from Sub-Saharan Africa and South Asia but is less common among immigrants from Middle-eastern countries [26]. In Sweden, hypertension has been found to be lower among immigrants of non-European origin [27], i.e. among immigrants with lower AF risks than Swedish-born individuals and higher among Finnish immigrants. In Bosnians, the risk of cardiovascular disorder (including CHD) has been shown to be increased in both women and men [28], which may partly explain their increased AF incidence as many risk factors are common for both conditions. In contrast to hypertension, the diabetes prevalence has been shown to be higher among immigrants of Middle Eastern origin, especially females, than immigrants from the Nordic countries [29]. This could possibly be linked to the increased AF incidence among Iraqi women. There are also contradictive findings in the literature regarding the relation between traditional cardiovascular risk factors and AF, e.g. a lower rate of AF among South Asians in the UK despite an adverse cardiovascular risk factor profile [14], and a higher AF risk among non-Hispanic whites in the US compared to non-Hispanic blacks, Chinese and Hispanics despite a lower prevalence of hypertension [11].

Dietary factors could be of importance for the development of AF [30]. For instance, the Mediterranean diet in the South European countries is associated with a lower risk of CHD [31] and intake of olive oil has also been associated with a lower AF risk [32]. In addition, high intake of fish with high levels of omega-3 fatty acids has also been found to prevent AF in some studies [33]. This association may possibly explain the lower AF incidence among Icelandic immigrants, even if the total evidence for this is inconclusive [34]. Otherwise, the lower AF incidence among immigrants from Iceland and Southern Europe could be attributed to their diet, which is high in marine food. Contradictory to this, incidence of AF on Iceland has increased during recent years [35]. Some of the other risk factors for AF, such as smoking [23] and high alcohol intake [30], seemed to be of minor importance to the AF incidence in the present study.

The healthy migrant effect, i.e. more healthy subjects tend to migrate [36], could be one important factor explaining the lower incidence of AF among first-generation immigrants, as more well-educated people migrate to Sweden from both Western and non-Western countries. However, in contrast to most Nordic neighbouring countries, immigrants from the Baltic countries, some Eastern European countries, and to some extent also Finland, tend to belong to the labour force group of immigrants. One Finnish twin study found that the twin who migrated to Sweden tended to have more cardiovascular risk factors than the non-migrant twin [37].

A novel finding in our study is the higher AF incidence in some groups with a high rate of war refugees to Sweden, e.g. from Bosnia during the 1990s and from Iraq after the turn of the millennium. Refugees may have experienced many stressful events, both before and during the migration, and stress has been shown to be associated with AF [38]. The concept of allostasis, i.e. the physiological response to acute stress [39] and of allostatic load, i.e. the accumulated side effects of life-course stress is thus of relevance; allostatic load is connected with the development of cardiovascular risk factors [40]. Thus, experiencing stressful events could possibly partially explain the higher AF incidence among male and female immigrants from Bosnia and female immigrants from Iraq. Another possibility is that refugees seek hospital care more frequently and will thus be more often examined and diagnosed with AF.

In addition to the more commonly recognised individual factors, socioeconomic factors are also of importance; lower family income and lower educational status have been shown to increase the risk of AF [41]. We also adjusted for neighbourhood-level SES as many immigrants, especially from non-Western countries, live in low SES neighbourhoods in urban areas. Living in low SES neighbourhoods is associated with an increased morbidity risk of AF-associated diagnoses [42], including cardiovascular health [16] and diabetes [43]. The mobility of individuals between different neighbourhoods is rather small [17].

The AF incidence pattern among second-generation immigrants differed in most cases only marginally compared to their Swedish-born counterparts with two Swedish-born parents, possibly due to acculturation, i.e. second-generation immigrants tend to adopt the lifestyle and health patterns of the host population over time and have a tendency to develop AF at the same rate.

A relevant question to ask is whether it is possible in the present study to recognise the finding in earlier studies that the health of immigrants tends to decline with years living in the new country [9, 10]. However, considering the diversity of the results in the different immigrant groups in the present study, as well as the diversity of the countries of origin of the immigrants, we judge that the results from such an analysis would be difficult to interpret.

This study has certain limitations. We had no data available on the type of atrial fibrillation (paroxysmal, persistent, or permanent). AF diagnoses were taken from the National Patient Register covering diagnoses from in-hospital patients and specialist open care, as data from primary care were not available to us. According to the data from Stockholm County this would cover 68% of all AF patients [5]. However, we consider a hospital diagnosis of AF to be of higher validity than a primary care diagnosis. Given that our focus was predominantly on cardiovascular co-morbidity and whether the relationship between neighbourhood SES and all-cause mortality is independent of cardiovascular comorbidity, we did not include other potential diagnoses associated with mortality such as presence of cancer or other non-cardiovascular medical prescriptions. In addition, we did not have access to multiple measures of individual SES. However, we adjusted our analyses for level of formal education, which is a commonly used proxy for individual SES [44]. As we explored multiple immigrant groups, there is a risk of mass significance due to multiple testing. We also performed a sensitivity analysis excluding subjects that had arrived in Sweden during the last five years. The statistical power to detect significant results also differed between the immigrant groups owing to varying sample sizes, and the power was lower among women, especially second-generation women.

Despite the limitations, one of the key strengths of this study is the linkage of clinical data (less than 1% missing data) from individual patients to national demographic and socioeconomic data. The clinical data were also highly complete; less than 2% of the total number of diagnoses were missing [45]. The comprehensive nature of our data made it possible to analyse men and women from all types of sociodemographic backgrounds.

In conclusion, we found an increased incidence of AF among certain immigrant groups, especially among immigrants from some war-torn regions, and a lower incidence among immigrant groups from countries with a traditionally healthy diet. From a clinical point of view, it is important to be aware of the increased incidence of AF in some immigrant groups in order to enable for a timely diagnosis, treatment and prevention of debilitating complications associated with AF, such as ischaemic stroke.

## Electronic supplementary material

Below is the link to the electronic supplementary material.

Supplementary material 1 (DOCX 20 kb)
