# Supplementary material for: Atrial fibrillation in immigrant groups: a cohort study of all adults 45 years of age and older in Sweden
Source: Eur J Epidemiol. 2017 Jul 12;32(9):785–96. doi: 10.1007/s10654-017-0283-6 (PMC5662664; doi:10.1007/s10654-017-0283-6)
Supplement: Supplementary file 1 — Supplementary material 1 (DOCX 20 kb) [file 10654_2017_283_MOESM1_ESM.docx]

| **Supplementary Table 1. Population and number of cases of atrial fibrillation diagnosis in the Swedish population used in the studies on first-generation and second-generation immigrants compared to Swedish-born, 1998-2012** | | | | | | | | | | | |
| --- | --- | --- | --- | --- | --- | --- | --- | --- | --- | --- | --- |
|  | First-generation analysis | | | | |  | Second-generation analysis | | | | |
| Country of origin | Population | |  | AF diagnosis | |  | Population | |  | AF diagnosis | |
|  | No | % |  | No. | % |  | No | % |  | No. | % |
| Sweden | 2,792,312 | 86.5 |  | 278,472 | 91.5 |  | 1,769,439 | 93.6 |  | 101,985 | 95.1 |
| Denmark | 30,137 | 0.9 |  | 2,032 | 0.7 |  | 13,771 | 0.7 |  | 578 | 0.5 |
| Finland | 147,444 | 4.6 |  | 97,73 | 3.2 |  | 36,692 | 1.9 |  | 1,558 | 1.5 |
| Iceland | 2,785 | 0.1 |  | 43 | 0.0 |  | 143 | 0.0 |  | 5 | 0.0 |
| Norway | 29,736 | 0.9 |  | 2,300 | 0.8 |  | 22,898 | 1.2 |  | 1,016 | 0.9 |
| France | 2,360 | 0.1 |  | 112 | 0.0 |  | 634 | 0.0 |  | 27 | 0.0 |
| Greece | 10,551 | 0.3 |  | 235 | 0.1 |  | 353 | 0.0 |  | 5 | 0.0 |
| Italy | 6,141 | 0.2 |  | 275 | 0.1 |  | 1,204 | 0.1 |  | 31 | 0.0 |
| Spain | 3,417 | 0.1 |  | 115 | 0.0 |  | 200 | 0.0 |  | 8 | 0.0 |
| Other Southern Europe | 1,834 | 0.1 |  | 57 | 0.0 |  | 44 | 0.0 |  | 0 | 0.0 |
| The Netherlands | 2,638 | 0.1 |  | 163 | 0.1 |  | 683 | 0.0 |  | 36 | 0.0 |
| UK and Ireland | 7,136 | 0.2 |  | 254 | 0.1 |  | 1,221 | 0.1 |  | 65 | 0.1 |
| Germany | 25,953 | 0.8 |  | 2,337 | 0.8 |  | 9,671 | 0.5 |  | 438 | 0.4 |
| Austria | 4,776 | 0.1 |  | 399 | 0.1 |  | 1,633 | 0.1 |  | 73 | 0.1 |
| Other Western Europe | 1907 | 0.1 |  | 109 | 0.0 |  | 495 | 0.0 |  | 19 | 0.0 |
| Bosnia | 6,033 | 0.2 |  | 325 | 0.1 |  | 46 | 0.0 |  | 1 | 0.0 |
| Yugoslavia | 23,767 | 0.7 |  | 1,319 | 0.4 |  | 1,093 | 0.1 |  | 26 | 0.0 |
| Croatia | 1,834 | 0.1 |  | 117 | 0.0 |  | 19 | 0.0 |  | 2 | 0.0 |
| Romania | 2,797 | 0.1 |  | 168 | 0.1 |  | 391 | 0.0 |  | 14 | 0.0 |
| Bulgaria | 914 | 0.0 |  | 47 | 0.0 |  | 65 | 0.0 |  | 2 | 0.0 |
| Other Eastern Europe | 760 | 0.0 |  | 43 | 0.0 |  | 4 | 0.0 |  | 0 | 0.0 |
| Estonia | 7,117 | 0.2 |  | 995 | 0.3 |  | 7,159 | 0.4 |  | 365 | 0.3 |
| Latvia | 1,289 | 0.0 |  | 179 | 0.1 |  | 1,278 | 0.1 |  | 74 | 0.1 |
| Poland | 12,381 | 0.4 |  | 793 | 0.3 |  | 3,248 | 0.2 |  | 100 | 0.1 |
| Other Central Europe | 4,563 | 0.1 |  | 355 | 0.1 |  | 1,217 | 0.1 |  | 48 | 0.0 |
| Hungary | 8,124 | 0.3 |  | 680 | 0.2 |  | 1,460 | 0.1 |  | 30 | 0.0 |
| Africa | 7,180 | 0.2 |  | 200 | 0.1 |  | 214 | 0.0 |  | 6 | 0.0 |
| North America | 8,066 | 0.2 |  | 518 | 0.2 |  | 7,543 | 0.4 |  | 393 | 0.4 |
| Chile | 6,188 | 0.2 |  | 129 | 0.0 |  | 62 | 0.0 |  | 1 | 0.0 |
| South America | 4,918 | 0.2 |  | 110 | 0.0 |  | 237 | 0.0 |  | 7 | 0.0 |
| Turkey | 7,058 | 0.2 |  | 357 | 0.1 |  | 216 | 0.0 |  | 2 | 0.0 |
| Lebanon | 2,031 | 0.1 |  | 64 | 0.0 |  | 8 | 0.0 |  | 0 | 0.0 |
| Iran | 8,062 | 0.2 |  | 228 | 0.1 |  | 64 | 0.0 |  | 1 | 0.0 |
| Iraq | 6,169 | 0.2 |  | 241 | 0.1 |  | 28 | 0.0 |  | 0 | 0.0 |
| Other Asian countries | 12,810 | 0.4 |  | 435 | 0.1 |  | 590 | 0.0 |  | 22 | 0.0 |
| Russia | 3,605 | 0.1 |  | 384 | 0.1 |  | 3,053 | 0.2 |  | 177 | 0.2 |
| Others | 21,959 | 0.7 |  | 124 | 0.0 |  | 3,777 | 0.2 |  | 98 | 0.1 |
| Total | 3,226,752 | 100.0 |  | 304,487 | 100.0 |  | 1,890,853 | 100.0 |  | 107,213 | 100.0 |

| **Supplementary Table 2. Incidence of (hazard ratio (HR) with 95% confidence intervals (95% CI)) AF in first-generation immigrants compared to Swedish-born, excluding immigrants moved in Sweden within 5 years*.** | | | | | | | |
| --- | --- | --- | --- | --- | --- | --- | --- |
|  | Men | | |  | Women | | |
|  | HR | 95% CI | |  | HR | 95% CI | |
| Sweden | 1 |  |  |  | 1 |  |  |
| **Nordic countries** | **0.83** | **0.81** | **0.85** |  | **0.97** | **0.95** | **0.99** |
| Denmark | **0.70** | **0.66** | **0.74** |  | **0.80** | **0.75** | **0.85** |
| Finland | **0.88** | **0.85** | **0.90** |  | **1.03** | **1.00** | **1.06** |
| Iceland | **0.31** | **0.20** | **0.48** |  | **0.51** | **0.33** | **0.79** |
| Norway | **0.87** | **0.82** | **0.93** |  | **0.89** | **0.84** | **0.94** |
| **Southern Europe** | **0.47** | **0.43** | **0.51** |  | **0.54** | **0.48** | **0.62** |
| France | **0.64** | **0.50** | **0.82** |  | 0.80 | 0.60 | 1.07 |
| Greece | **0.39** | **0.33** | **0.45** |  | **0.41** | **0.33** | **0.52** |
| Italy | **0.53** | **0.46** | **0.60** |  | **0.48** | **0.38** | **0.61** |
| Spain | **0.46** | **0.36** | **0.59** |  | 0.76 | 0.57 | 1.03 |
| Other Southern Europe | **0.39** | **0.28** | **0.55** |  | **0.61** | **0.39** | **0.94** |
| **Western Europe** | **0.76** | **0.73** | **0.80** |  | 0.95 | 0.91 | 1.00 |
| The Netherlands | **0.67** | **0.55** | **0.81** |  | 0.85 | 0.66 | 1.10 |
| UK and Ireland | **0.60** | **0.51** | **0.70** |  | **0.60** | **0.49** | **0.74** |
| Germany | **0.80** | **0.75** | **0.85** |  | 1.00 | 0.95 | 1.06 |
| Austria | **0.82** | **0.72** | **0.93** |  | 1.00 | 0.85 | 1.17 |
| Other Western Europe | **0.70** | **0.55** | **0.89** |  | **0.72** | **0.53** | **0.99** |
| **Eastern Europe** | **0.76** | **0.72** | **0.81** |  | 0.94 | 0.87 | 1.01 |
| Bosnia | **1.47** | **1.26** | **1.71** |  | **1.53** | **1.22** | **1.93** |
| Yugoslavia | **0.71** | **0.66** | **0.76** |  | **0.90** | **0.82** | **0.98** |
| Croatia | **0.74** | **0.59** | **0.93** |  | 1.06 | 0.78 | 1.43 |
| Romania | **0.73** | **0.60** | **0.88** |  | **0.74** | **0.57** | **0.97** |
| Bulgaria | **0.66** | **0.46** | **0.96** |  | 0.97 | 0.60 | 1.56 |
| Other Eastern Europe | **0.61** | **0.42** | **0.89** |  | 0.95 | 0.54 | 1.67 |
| **Baltic countries** | 1.07 | 0.99 | 1.16 |  | 1.08 | 0.99 | 1.17 |
| Estonia | 1.05 | 0.96 | 1.15 |  | 1.10 | 1.00 | 1.20 |
| Latvia | 1.18 | 0.97 | 1.44 |  | 0.97 | 0.78 | 1.21 |
| **Central Europe** | **0.75** | **0.70** | **0.80** |  | **0.87** | **0.81** | **0.93** |
| Poland | **0.73** | **0.66** | **0.80** |  | **0.85** | **0.77** | **0.94** |
| Other Central Europe | **0.78** | **0.68** | **0.89** |  | **0.81** | **0.69** | **0.96** |
| Hungary | **0.76** | **0.69** | **0.83** |  | 0.93 | 0.82 | 1.05 |
| **Africa** | **0.48** | **0.41** | **0.56** |  | **0.57** | **0.40** | **0.82** |
| **North America** | **0.71** | **0.62** | **0.80** |  | 0.90 | 0.80 | 1.01 |
| **Latin America** | **0.37** | **0.31** | **0.44** |  | 0.48 | 0.39 | 0.59 |
| Chile | **0.34** | **0.27** | **0.42** |  | **0.49** | **0.37** | **0.64** |
| South America | **0.43** | **0.33** | **0.54** |  | **0.48** | **0.35** | **0.66** |
| **Asia** | **0.48** | **0.44** | **0.51** |  | **0.77** | **0.70** | **0.85** |
| Turkey | **0.53** | **0.46** | **0.61** |  | 0.89 | 0.76 | 1.04 |
| Lebanon | **0.43** | **0.32** | **0.58** |  | **0.62** | **0.40** | **0.95** |
| Iran | **0.40** | **0.34** | **0.47** |  | **0.71** | **0.56** | **0.91** |
| Iraq | **0.53** | **0.42** | **0.66** |  | 1.11 | 0.79 | 1.56 |
| Other Asian countries | **0.49** | **0.43** | **0.55** |  | **0.68** | **0.58** | **0.80** |
| **Russia** | 0.98 | 0.84 | 1.13 |  | 0.92 | 0.79 | 1.06 |
| *: Adjusted for age, region of residence in Sweden, educational level, marital status, neighbourhood deprivation, and comorbidities  Significant HRs (with 95% CIs) are marked by bold | | | | | | | |
